# A Review on Phyto-Therapeutic Approaches in Alzheimer’s Disease

**DOI:** 10.3390/jfb14010050

**Published:** 2023-01-16

**Authors:** Mehtap Sahiner, Aynur Sanem Yilmaz, Buket Gungor, Nurettin Sahiner

**Affiliations:** 1 Department of Bioengineering, Faculty of Engineering, Canakkale Onsekiz Mart University, Terzioglu Campus, Canakkale 17100, Turkey; 2 Department of Chemistry, Canakkale Onsekiz Mart University, Terizoglu Campus, Canakkale 17100, Turkey; 3 Department of Pharmacology, School of Medicine, Canakkale Onsekiz Mart University, Terzioglu Campus, Canakkale 17100, Turkey; 4 Nanoscience and Technology Research and Application Center (NANORAC), Canakkale Onsekiz Mart University, Terzioglu Campus, Canakkale 17100, Turkey; 5 Department of Ophthalmology, Morsani College of Medicine, University of South Florida, 12901 Bruce B. Downs Blv., MDC 21, Tampa, FL 33620, USA; 6 Department of Chemical, Biological, and Materials Engineering, University of South Florida, Tampa, FL 33620, USA

**Keywords:** phytocompounds, neurodegenerative diseases, metal chelating, enzyme inhibitors, polyphenolics

## Abstract

Neurodegenerative diseases occur due to progressive and sometimes irreversible loss of function and death of nerve cells. A great deal of effort is being made to understand the pathogenesis of neurodegenerative diseases. In particular, the prevalence of Alzheimer’s disease (AD) is quite high, and only symptomatic therapy is available due to the absence of radical treatment. The aim of this review is to try to elucidate the general pathogenesis of AD, to provide information about the limit points of symptomatic treatment approaches, and to emphasize the potential neurologic effects of phytocompounds as new tools as therapeutic agents for disease prevention, retardation, and therapy. This survey also covers the notable properties of herbal compounds such as their effects on the inhibition of an enzyme called acetylcholinesterase, which has significant value in the treatment of AD. It has been proven that phytopharmaceuticals have long-term effects that could protect nervous system health, eliminate inflammatory responses, improve cognitive damage, provide anti-aging effects in the natural aging process, and alleviate dementia sequelae. Herbal-based therapeutic agents can afford many advantages and can be used as potentially as new-generation therapeutics or complementary agents with high compliance, fewer adverse effects, and lower cost in comparison to the traditional pharmaceutical agents in the fight against AD.

## 1. Introduction

Alzheimer’s Disease (AD) is a progressive neurodegenerative disease characterized by the loss of function of neurons and their consequent death [1]. Although AD is not a natural consequence of aging, it is generally seen in the elderly population [2]. The main symptoms encountered in Alzheimer’s disease and Alzheimer’s-related dementia are called neuropsychiatric symptoms (NPS) [2]. Although depression takes the first place, schizophreniform or paranoid psychosis is one of the other most common types of these symptoms [3]. Describing overt syndromes in patients can become quite complex, as these NPSs may overlap into clusters of symptoms [2]. However, some of the AD syndromes have been found to be quite consistent in AD in systematic studies performed on various populations [4,5]. These common syndromes include mild onset but progressive cognitive dysfunction, apathy, sleep disorders, psychosis, and agitation [6]. Alzheimer’s patients also face many problems such as perception disorders, problems in learning skills and memory, slips of the tongue, executive dysfunction, regression in multitasking skills, and loss of confidence. Depending on the severity of dementia seen in patients, individuals become dependent on special care.

Several different diagnostic criteria have been published, examining cerebrospinal fluid biomarkers and imaging techniques for the classification of AD [7]. According to the Mini-Mental State Examination score (a short screening tool that expresses the severity of the condition in neurological disorders such as AD and dementia by grading cognitive impairment) [8] and the severity of the disease sequelae, AD can be examined in subgroups as moderate or severe [9]. In addition, although there are different opinions on the subject, the term pre-clinical AD is generally used for cases with AD pathology but no dementia, regardless of whether it has passed into the symptomatic stage [10]. Deterioration in memory function and difficulties in daily activities could occur in the advanced stages of AD progression, as well as they may start long before physical symptoms [11,12].

As in many neurodegenerative or chronic diseases, it is necessary to have knowledge about the role of certain individual differences such as gender, age, and race in susceptibility to the disease in AD. Based on this information, the creation of new therapeutic strategies to prevent disease will be more consistent, reliable, and faster [13,14,15].

## 2. Epidemiology of AD

AD is seen as the predominant cause of dementia and accounts for more than two-thirds of it in the elderly population [16,17]. Therefore, the prevalence of AD seriously threatens public health [18]. In 2005, experts commissioned by AD International reached some conclusions on the prevalence and estimated incidence of dementia in 14 World Health Organization regions, based on the current epidemiological data at that time [13,14]. According to their data, 24.2 million people lived with dementia at that time, with about 4.6 million new cases emerging each year [13]. According to the report published in 2015, the number of dementia patients at that time increased to 50 million globally [15]. The prevalence appears to double every five years after the age of 65 [16]. On the other hand, in a meta-analysis conducted in previous years, it was suggested that the incidence of AD, which increases sharply in individuals over 65 years of age, increased more slowly or did not increase after the age of 90 [19].

In a national study, it was found that the incidence of AD in individuals aged 50 years and older rose roughly from 5.63 to 8.18 per 1000 population between 2005 and 2010 [20]. According to the results of a meta-analysis conducted in 2017, the prevalence of AD in Europe was estimated at 5.05% [21]. The incidence of AD was reported as 11.08% per 100 person-years in Europe [21]. It was stated that both prevalence and incidence were higher in women than in men and generally increased with age [21].

There are many studies examining the incidence rate of AD, but unfortunately, there are limitations in these estimations, such as determining the age stage, at which the pathological process starts, and defining individuals who do not have the disease [14]. In some epidemiological studies, data inferences and estimates lacked precision due to significant statistical heterogeneity [22,23,24,25]. Nevertheless, the existing studies are a baseline for new studies on preventive and disease-modifying interventions and therapeutic approaches in AD, and some important predictions and implications remain important [26,27,28].

**Figure 1:**
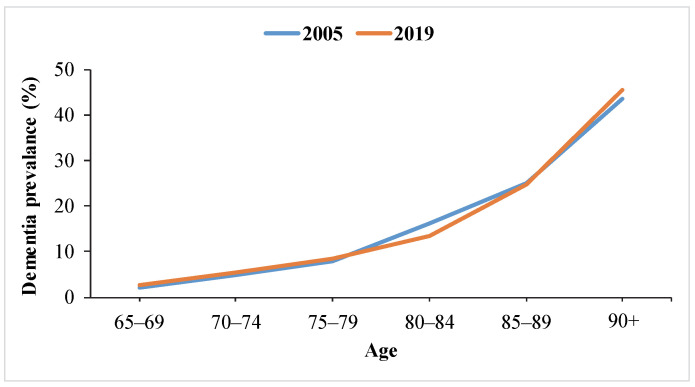
Roughly estimated global dementia prevalence by age in 2005 and 2019.

Figure 1 represents the approximate dementia occurrence as a percentage by age in 2005 and 2019, which was created by using related prevalence and incidence rate results inferred from three different studies [1,2,3].

As seen in Figure 1, according to the data obtained with an interval of about 15 years, the prevalence of dementia in the world has been determined to be approximately 25% in the age group of 85–89 and more than 40% over the age of 90. Therefore, it can be inferred that the prevalence of dementia is mostly seen in the population aged 85 and over and can reach as high as 30–50%.

The prevalence and incidence rates calculated in different periods are expected to double by 2050, especially due to the increase in the number of individuals aged 65 and over [29]. Estimates show that AD will emerge as an even more serious public health problem in the near future [1,12].

When radical treatment or complete recovery is not possible, only symptomatic treatment approaches are available. Novel therapies are still being tried and understanding the histopathology of the disease is one of the most important points. In the next section, the etiological factors, and histopathological changes in the pathology of AD will be explained.

## 3. Etiology and Pathogenesis of AD

### 3.1. Etiology of AD

#### 3.1.1. Genetic Predisposition to AD

There are genetic and environmental risk factors in the pathology of AD. It has not been fully elucidated how genetic factors are involved in the etiology [6,11,30]. It was reported that in a very low (1–5%) rate of cases, genetic abnormalities in chromosomes caused AD in the early stages [2,17,31,32]. Mutations in genes encoding certain protein precursors are also among the genetic deficits in AD pathology [2,32]. The strongest common genetic variant for typical late-onset AD seen after 65 years of age is apolipoprotein E (apoE), a three-allele polymorphism. Of these alleles, ε3 is considered a neutral allele, ε4 is the high-risk allele, and ε2 is considered a protective allele [17]. The ε4 type allele of the apoE gene was identified as a very common genetic risk factor for sporadic late-onset AD [33]. Unlike the gene associated with early-onset familial AD, this allele is not determinative. However, it was noted that the risk of developing AD was tripled if one copy of the ε4 allele was present, and eight-fold if two copies were present [34].

The presenilin gene is a member of the gamma-secretase family, which is thought to play an important role in the formation of amyloid beta from the precursor protein [35]. Alzheimer’s patients may encounter Alzheimer’s phenomenon from family history at an early age due to the susceptibility to mutations that may occur in this gene [36]. Triggering receptor expressed on myeloid cells 2, also known as TREM-2, is a cell surface receptor that mediates phagocytic clearance of neuronal debris [31]. It was determined that a rare mutation in the gene encoding this protein led to the inability to clear β-amyloid proteins from the central nervous system (CNS) [37]. It was reported that the increased accumulation of non-cleared proteins caused the pathogenesis to become more intense and the neurodegenerative condition to become more severe [31,37].

As mentioned in the previous paragraph, genetic risk factors in the development of AD are diverse, and they may not be fully associated with the disease. Moreover, AD cases due to genetic abnormalities represent a very small percentage of all cases. On the other hand, approximately 90–95% of all Alzheimer’s cases are late-onset [32], and thanks to histopathological evaluations in the clinic, certain biomarkers could be closely associated with the pathogenesis of AD [11,38].

#### 3.1.2. Non-Genetic Factors in the Pathogenesis of AD

The distinctive prominent protein conformational disease definition is also used for AD. The reason for this, if genetic factors are put aside, is that normally soluble proteins reach differentiated conformations due to aging, external factors, and aggregation as a result of misfolding [31]. In addition, although aging-related changes are involved in the pathology of dementia and AD, these two neurological disorders are not a natural consequence of the ‘aging’ process [20].

Understanding the pathogenesis of the disease and risk factors is essential, as Alzheimer’s and many other neurodegenerative diseases are attempted to be treated by targeting specific regions in the brain, specific biomarkers, and certain pathological pathways.

#### 3.1.3. Relationship between Chronic Diseases and AD Progression

- Cerebrovascular Disease–AD relationship.

Non-genetic risk factors could be related to the co-existence of another chronic or inflammatory disease or the sequelae of a previous disease [31]. Pendlebury and Rothwell estimated the incidence of new-onset dementia after the first stroke to be almost 7%, based on data from related cohort studies [39]. Post-stroke dementia has been strongly associated with the occurrence of multiple lesions and the incidence of stroke-related complications [17,39]. One of the mechanisms responsible for ischemic stroke causing permanent cognitive impairment is thought to be the destruction of the brain parenchyma together with hippocampal atrophy [40].

The phenomenon of stroke in individuals has also been associated with a condition called amnestic syndrome, in which cognitive functions are preserved but acquired information, facts, and experiences cannot be stored in memory [40]. Cerebral senile plaque formation was seen more in individuals with good cognitive functions but with critical coronary artery disease than in individuals without any heart disease. Moreover, increased neurofibrillary tangles have been identified in asymptomatic young individuals without dementia or clinical AD, and these symptoms have been of great benefit in early diagnosis [41].

- Cardiovascular disease–AD relationship.

It was reported that untreated high blood pressure increased the risk of vascular dementia and AD in middle-aged individuals [42]. It was suggested that middle-aged hypertensive cases were associated with AD-specific neuropathological changes [43]. Launer et al. (2000) found that high blood pressure increased the risk of dementia in patients whose blood pressure was not regulated by antihypertensive agents, and that this risk did not, however, increase when it was regulated [42].

The presence of high serum cholesterol concentration in addition to increased systolic blood pressure significantly increases the risk of AD in advanced age [41]. There are several reasons why cholesterol is involved in the pathogenesis of AD. First, the cholesterol-carrier protein ApoE promotes amyloid plaque aggregation. Second, the balance between free cholesterol and cholesterol esters modulates the formation of these plaques. Moreover, cholesterol poses a serious risk of dementia by causing permanent vascular damage via ischemic pathways [11,44,45]. Therefore, like hypertension, hyperlipidemia has taken its place among these risk factors [17,42,43].

With the onset and progression of AD, blood pressure tends to decrease for reasons attributable to atherosclerosis, weight loss, and autonomic regulation of blood flow. Similarly, cases of abnormal blood pressure have been reported in the advanced stages of dementia. However, since the risk of cardiac infarction in hypertension patients increases with aging, the onset of AD may not be seen at all; therefore, it is difficult to explain the relationship between AD and hypertension [15,30,41].

Nonetheless, many cohort studies showed that high blood pressure and cholesterol may be involved in the pathogenesis of AD, and that new strategic methods for cardiovascular risk factors could be used in the symptomatic treatment of AD [31,41,42].

- Cardiovascular disease–AD relationship.

Insulin can cross the blood–brain barrier and is produced in the brain. There are different approaches regarding the function of insulin in the brain [17]. Impaired central nervous system, insulin metabolism, and insulin dysregulation are known as risk factors for AD. One view is that hyperinsulinemia associated with type 2 diabetes directly impairs protein clearance and causes accumulation in the brain, as it prevents protein accumulation thanks to its insulin-degrading enzyme [10,46,47]. Another view is that insulin controls tau phosphorylation in the brain. Peripheral hyperinsulinemia down-regulates insulin uptake across the blood–brain barrier due to supersaturation above physiological levels. This results in the downregulation of the insulin-degrading enzyme involved in amyloid clearance in the brain. Moreover, it was thought that decreased insulin signaling or response may induce the formation of neurofibrillary tangles through effecting glycogen synthase kinase 3 (an enzyme that modulates various intracellular signaling pathways such as energy metabolism and neuronal cell development) activity [10,46,47,48].

#### 3.1.4. Other Non-Genetic Risk Factors in AD

Individuals who have had a traumatic brain injury and especially those who carry the ApoE-E4 allele (an important genetic factor) have been found to have a higher risk of dementia [49]. Post-mortem and experimental studies determined that intraneuronal tau pathology increased even in young patients after traumatic brain injury [50]. β-amyloid protein deposition occurs rapidly shortly after traumatic brain injury, while the formation of neurofibrillary tangles occurs in the chronic phase of the disease [50,51].

**Figure 2:**
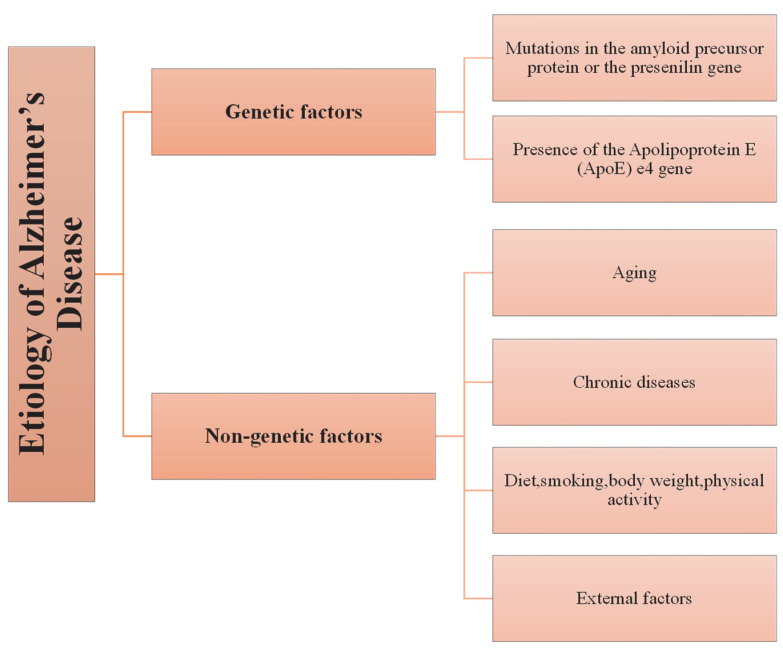
Schematic presentation of genetic and non-genetic factors involved in the etiology of AD.

In addition to these, factors that may cause chronic diseases, such as smoking, body weight, diet, and sedentary life, pose an important risk for AD in the early period [41,43,47]. For example, a meta-analysis found an association between obesity and the risk of AD, which may be attributable to insulin resistance [52]. Smoking, on the other hand, is known to cause an increase in oxidative stress, which may play an essential role in AD; although, nicotine improves attention and information processing by increasing the release of acetylcholine [53]. Genetic and non-genetic risk factors in AD are summarized in Figure 2.

AD risk-modifying factors can be modulated by changing patients’ lifestyles (used as a prophylactic change or prevent disease progression agent), early diagnosis, and intervention with therapeutic agents [17]. Promising results are obtained when attempting to modulate risk factors. For example, it was determined that antioxidant polyphenolic compounds in the Mediterranean diet model reduced the risk of AD [17]. On the other hand, the risk of inflammation and the occurrence of cerebrovascular changes in Type 2 diabetes patients are the factors that significantly increase the risk of AD formation [6].

### 3.2. Pathogenesis of AD

Although certain biomarkers and pathophysiological changes are associated with the onset of AD, there are still unexplained points in the pathogenesis of AD [17]. There are different theories such as cholinergic hypothesis and excitotoxicity in the pathogenesis of AD, and different approaches were developed for each pathway. Among these, the amyloid cascade hypothesis, in which amyloid-beta and tau proteins are involved in the pathological process, was clarified more clearly than the others [25,31,54]. In the studies carried out by Jack et al. (2018) within the scope of the National Institute on Aging and Alzheimer’s Association (NIA-AA), the Research Framework was stated as follows:

Although different factors are involved in the emergence of the disease and some histopathological findings have not been fully elucidated, β-amyloid plaques and neurofibrillary tau deposits are specific biomarkers for AD [55].

β-amyloid peptides(Aβ) are derived from the enzymatic reactions of amyloid precursor proteins (APP), which are transmembrane glycoproteins. APP can be altered by beta- and gamma-secretases to produce insoluble fibrils. The accumulation of these insoluble fibrils causes amyloid pathogenesis [31]. The first biomarker specific to AD is the extracellular accumulation of these Aβ in the brain parenchyma and cerebral blood vessel walls [56].

Tau proteins are axonal proteins involved in the organization and stabilization of microtubules that make up the cytoskeleton and function by binding to tubulin monomers. These proteins are predominantly expressed in neurons [38]. Therefore, the function of these proteins is important in the preservation of cell morphology and the formation of nucleolar structure.

At the stage of oligomerization, Aβ diffuses into synaptic clefts, interfering with synaptic signaling. When polymerized into insoluble amyloid fibrils, it causes activation of kinases that lead to hyperphosphorylation of tau protein [57]. In addition to aberrant phosphorylation, misfolding and aggregation of these proteins are followed by the formation of tau-containing neurofibrillary tangles (NFTs), which transform into double-helix filament forms [38]. A protease-resistant tau protein core emerges in AD, and this formation initiates the process of binding more tau molecules and abnormal tau aggregation. Once tau aggregation begins, the function of the endosomal-lysosomal pathway used for the clearance of proteolytically stable oligomers is compromised. Because of their being insoluble, once neurofibrillary tangles are formed and settled, they are almost impossible to remove [11]. Therefore, the presence of hyperphosphorylated or ‘mutant’ tau proteins has been identified as another determinant biomarker in AD.

Mutant tau aggregates are known to cause toxic function as well as destabilization [11,36,56]. Aggregates that cannot be cleared and accumulated in certain areas of the brain cause a decline in cognitive function and synaptic degeneration [38,58].

**Figure 3:**
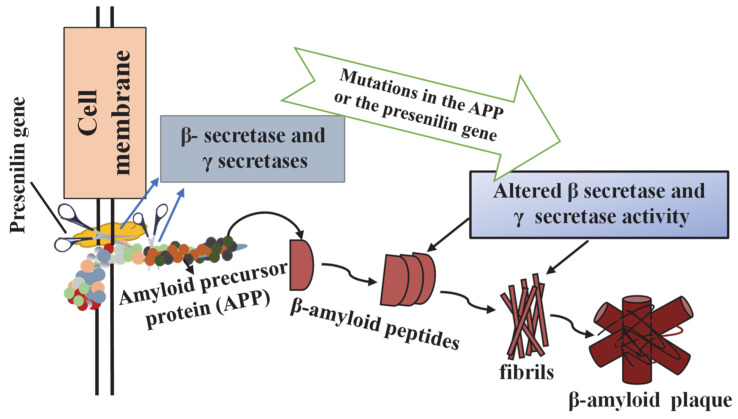
Demonstration of β-amyloid plaque formation. Adapted from [62].

Another type of interaction and integration of extracellular Aβ with enlarged (dilated) neurites containing pathological tau aggregates is called senile (neurotic) plaques [32]. Senile plaques are seen as disseminated in all cerebral cortex and subcortical structures. The principal component in senile plaques is Aβ0, and the two major forms of Aβ are the 40-residue Aβx-40 and the 42-residue Aβx-42, which are more prone to form amyloid deposits [59]. Although not fully elucidated, the Aβ has such an important role in the pathogenesis that the density of plaques and the level of Aβ have also been proven to be a measure of the severity of dementia [60]. Aβ plaques initially develop in the basal, temporal, and orbitofrontal regions of the brain, and in later stages progress along the basal ganglia. In critical situations, Aβ is also found throughout the lower brainstem and cerebellar cortex. This Aβ concentration triggers the tangle formation of tau proteins in certain regions. In the critical stage, this formation spreads to the hippocampus and neocortex [61]. As a result of the accumulation of plaques and tangles, microglia uptake develops, surrounding the plaques and promoting microglial activation and local inflammatory response. In addition to neurofibrillary tangles and senile plaques, inflammation, synapse losses, cortical cholinergic innervation, and losses in other neurotransmitter systems constitute the components of AD neuropathology [57]. The level of Aβ did not significantly increase in demented elderly schizophrenic patients who did not show Alzheimer’s-related histopathology [1]. The fact that it does not increase similarly in a different neuropathological disorder showing cognitive decline has proven the specificity of Aβ for AD [1]. The Aβ deposition is upstream of tau pathology. In addition, the consistent Aβ deposition occurs years before severe tau peptide pathology occurs, and tau peptide mutations cause tauopathy and induce neurodegenerative disease [32]. The demonstration of Aβ plaque formation is given in Figure 3.

Figure 3 shows the process by which Aβ produced from amyloid precursor protein (APP) misfolds into insoluble oligomers and subsequently accumulates to form Aβ plaques.

**Figure 4:**
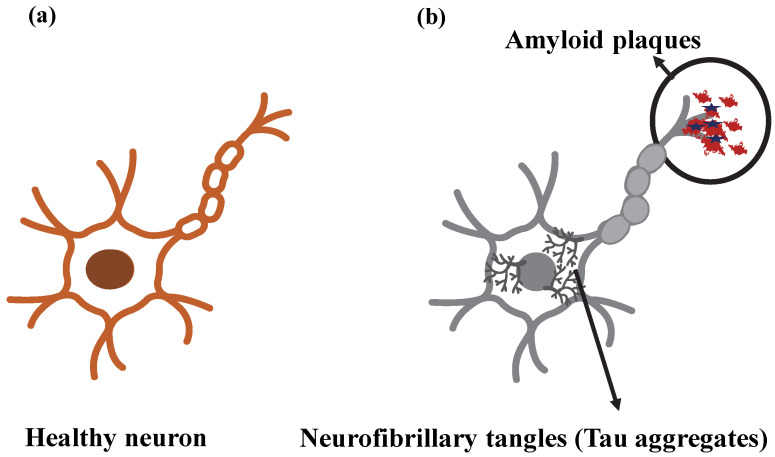
Representation of (**a**) healthy neuron and (**b**) neuron showing Alzheimer’s disease (AD) pathological markers.

Different mechanisms play a role in the accumulation of Aβ in the brain, leading to neurotoxicity. Accumulation of Aβ in cerebral arterioles is called “amyloid angiopathy” and can cause cerebral lobar hemorrhages. One idea is that microglial and astroglial cells are activated because of protein aggregation. Inflammation occurs with the secretion of cytokines from microglial cells activated by Aβ and inflammatory stimuli, activation of acute phase reactants, and complement [57,63,64]. Proinflammatory cytokines and neurotoxins released by the inflammatory response can cause or exacerbate neuronal damage. Once the neurotic plaque has formed, a secondary cascade of inflammation, excitotoxicity, and possibly apoptosis mediates further damage. Permanent damage, on the other hand, is the result of Aβ accumulation causing increased apoptosis by disrupting intercellular communication, resulting in tissue dysfunction. In addition, amyloid neurotoxicity and transsynaptic degeneration are also thought to play a role in cell death [63,64,65]. Protein deposits and plaques formed in AD are shown in the nerve cell in Figure 4.

Figure 4 reflects the localization of two key markers specific to Alzheimer’s pathogenesis on the neuron. The data obtained from the brain tomography of the patients revealed that the aggregates of Aβ and neurofibrillary tangles had a specific distribution pattern and different densities [66].

The two main pathological changes that are specific to AD can basically be handled in this way. In the previous sections, we aimed to explain the changes in the brain in the pathogenesis of AD in a clear and informative way.

As in all other chronic diseases, early diagnosis studies in AD are essential in the treatment of the disease and in alleviating symptoms. A biological definition rather than a syndrome-based definition seems to be more logical in the clinical approach to AD [55,67,68].

Prognostic biomarkers have an undeniable place in the clinical approach to Alzheimer’s disease to detect and evaluate the decline in cognitive function. With the functional assessment questionnaire, cognitive function decline can be measured with the activities of the daily living parameter. Patients with a high score in this evaluation have advanced cognitive impairment [69]. In addition, it is vital to control and detect brain amyloidosis in cognitively normal individuals because the rate of Aβ positive (in terms of risk assessment) in cognitively normal individuals was reported to be 30% or more in individuals over 80 years of age [70].

**Figure 5:**
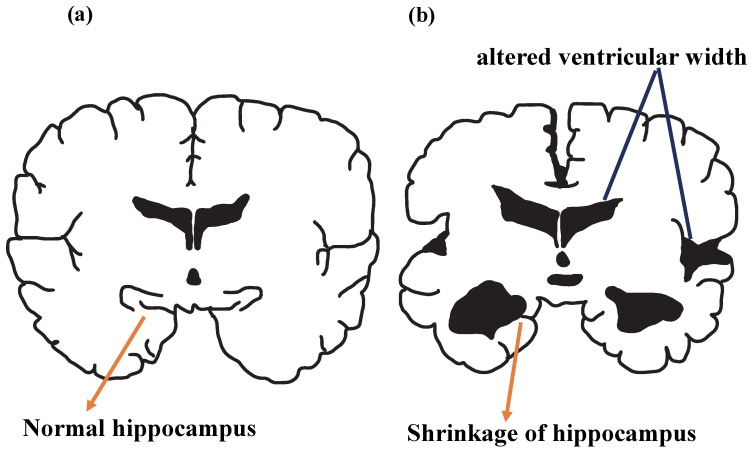
Illustration of (**a**) normal brain and (**b**) AD brain.

By using various imaging techniques, such as amyloid positron emission tomography, changes in the brain can be detected and biologically targeted by neuropathological examination. Among these changes, amyloid biomarkers were determined as the earliest detectable neuropathological condition in AD progression [55,67,68]. Cerebral atrophy detected in magnetic resonance imaging (MRI) scans of Alzheimer’s patients is shown in Figure 5a,b for normal and AD brains [6].

After the pathogenesis of AD occurs, the vascular system cannot provide sufficient blood and nutrients to the brain, and in this case, the brain is deprived of the energy necessary for its function. Failure of microglial cells to clear debris leads to chronic inflammation. As a result, neurons lose their ability to communicate and permanent neuron damage occurs. Figure 5 represents hippocampus shrinkage, a complication of this pathological process. It is known that hippocampal atrophy can cause memory loss, impaired decision-making, speech difficulties, and some AD-specific symptoms over time [71].

## 4. Conventional Approaches to AD

Although it is controversial to use the term conventional treatment (because this radical treatment is not currently available), there are certain groups of drugs that have been involved in the symptomatic treatment of AD for many years. In addition to these, there are promising novel therapies that are still in the trial phase, but their safety and efficacy have not been proven [26,31].

### 4.1. The Role of Acetylcholinesterase Inhibitors

One of the main approaches in symptomatic treatment is to reduce synaptic acetylcholine degradation by using AChE inhibitors (AChEI) such as donepezil, tacrine, rivastigmine, and galantamine. Although tacrine is the first cholinesterase inhibitor, its use has been restricted due to its liver toxicity [72]. As acetylcholine breakdown is blocked in synapses, its activity and cholinergic transmission increase [73]. AChEI presented successful results in AD cases of varying severity but showed the most activity in the mild to moderate group [5,74]. In a 6-month, multicenter, and double-blind study in patients with mild to moderate AD, galantamine was found to be safe, and significantly improved cognition [75]. There is no superiority among these drugs, and the appropriate drug is selected based on the dosage form, route of administration, and tolerance of the patient [11,72].

In addition to AChEI, cholinergic agonists of acetyl precursors are also included in the treatment. Similarly, the aim here is to target amyloid structure by increasing cholinergic neurotransmission [25,76]. For example, nicotinic receptor agonists (a group of drugs that mimic the action of acetylcholine) were also found to increase cognitive function by preventing nerve cell degeneration [77].

Unfortunately, AChEI group drugs do not provide any benefit in a significant part of patients, and most of them cause serious side effects originating from the gastrointestinal system [2,75,78]. The most common side effects are nausea, vomiting, loss of appetite, fatigue, drowsiness, insomnia, and muscle cramps; more serious ones are confusion, arrhythmia, and bradycardia. In addition, the use of AChEI group drugs is limited in individuals with a history of peptic or duodenal ulcers, and attention should be paid to such patient groups. Agitation and an acute worsening of cognition can be observed when starting medication with this group of drugs. In this case, it is not considered appropriate to continue the drug [54]. It is generally expected that roughly one-third of patients cannot tolerate a cholinesterase inhibitor due to the exhaustion of side effects [75,78]. Although in 1993, tacrine became the first AChEI to be approved by the FDA, the high incidence of side effects, including hepatotoxicity, has led to its abandonment [79].

### 4.2. Memantine

Memantine, one of the antagonists of the N-methyl-D-aspartate receptor (NMDA-receptor family involved in learning and memory), is used to alleviate dementia symptoms by preventing excitotoxicity [11]. In addition to its neuroprotective effect, memantine is thought to restore the function of damaged neurons [54]. Since there is no known interaction with each other, the combined use of AChEI and memantine has been tried and behavioral symptoms have been found to improve in moderate-to-severe AD [80]. The addition of memantine to donepezil monotherapy may also be promising in mid-stage AD. This combination has reduced the 24-week decline in cognitive status, provided a reliable and tolerable effect, and given statistically significant results [81].

### 4.3. Other Treatment Approaches in AD

#### 4.3.1. Immunotherapy-Based Approaches to AD

Although it has been shown that immunotherapy can improve pathological symptoms in AD [4,82], this therapy, unfortunately, does not have a clear role in the treatment. The reason for this can be explained as follows: Tau proteins are intracellular proteins and antibodies must cross the blood–brain barrier and neuronal plasma membrane to influence these proteins. On the other hand, it is possible that tau in the extracellular space may be targeted by antibodies and additional defense mechanisms may be activated [56]. In a study using transgenic mice (2003), immunization was found to prevent the development of Alzheimer’s-like neuropathology [11]. Immunization tests with Aβ on humans could not progress due to safety concerns. Studies are still ongoing to create a safe and highly effective immunotherapy method [82].

#### 4.3.2. The Use of Antipsychotic and Antidepressant Drugs in AD

The vast majority of therapeutic approaches to AD are aimed at eliminating or reducing the levels of Aβ plaques [54]. Some antipsychotic agents, such as haloperidol, are used in the treatment by inhibiting the production of Aβ [1]. However, in cases where dementia accompanies AD, only moderate effects can be obtained, regardless of the type of the antipsychotic drug used [25].

Studies evaluating the precision and reliability of the effects of antidepressants against the sequelae of AD are few. Although the use of some antidepressants to relieve depression in dementia patients is positive, tricyclic antidepressants can worsen the confusion, and their use is not appropriate [25,83].

#### 4.3.3. Effects of Statins in AD

Cholesterol dysregulation and vascular damage were also thought to play a role in the pathogenesis of AD [34,41]. Cholesterol dysregulation causes interactions with ApoE, which has an important role in AD and is known to cause vascular damage. Statins can reduce Aβ production by shifting the balance away from beta- and gamma-secretases. It is also among the thoughts that they provide healing thanks to their anti-inflammatory effects [61,78,84]. In a retrospective case-control study (2000), individuals, who used at least one dose of statin group drugs in the previous 6 months, were 70% less likely to develop AD than control groups [84]. On the other hand, the same result was not observed when a different lipid-lowering drug group was used [84]. However, statins are a large group of drugs, and not every drug in the group was found to be effective in AD [11,85].

#### 4.3.4. The Role of Vitamin Supplements in AD Therapy

Nutrients, such as Vitamin E, vitamin C, β-carotene, and flavonoids, which have antioxidant properties against oxidative stress, can also be included in the treatment [86]. In a retrospective study (2002), the risk of AD was reduced by 34% and 43%, respectively, when vitamin C and vitamin E were taken in more than certain amounts, compared to groups that received less supplementation [87]. Although there was no exact causal relationship, it was observed that folate deficiency in mice sensitized neurons to amyloid toxicity. Therefore, regulation of folate levels in the body may be beneficial in AD [88].

## 5. Limiting Factors in Current Approaches to AD

**Figure 6:**
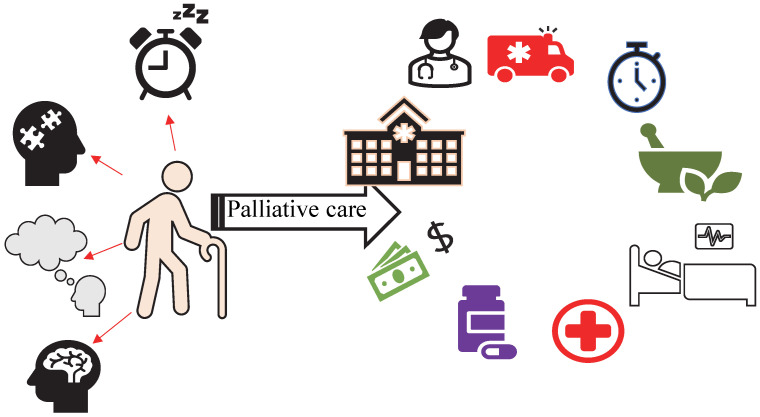
Long-term burden of symptomatic treatment approaches on health services in Alzheimer’s patients.

Benefiting from early diagnosis services is very beneficial in neurological disorders, as in many other chronic diseases. However, in diagnosing Alzheimer’s disease, although clinical symptoms, the patient’s family history, and other findings are evaluated, it may be too late to diagnose because some symptoms are not specific to this disease [89]. Moreover, although some neurodegenerative disorders, such as AD, show similar patterns among patients, the underlying cause of their occurrence is not always clear. At this point, there is a need for individualized treatments specific to patients. However, symptomatic treatment agents used in AD therapy and treatment cannot achieve sufficient success in this ‘personalization’ phase [25]. The long-term burden of symptomatic treatment approaches on health services in Alzheimer’s patients is described in Figure 6.

The prevalence and mortality of AD are increasing, and the inability of treatments to be more than palliative is a heavy burden on healthcare [14,18]. Preventive treatments are an ideal option for neurodegenerative diseases in general. In addition, although their efficacy has not been proven, these newer therapeutic agents may have fewer adverse effects on the body. In contrast, most of the prophylactic treatments currently in use are long-term, tiring, and costly. For this reason, the treatment should be modified considering the burden it will create on the patient, the patient’s relatives, and healthcare professionals. In addition, there is a need for physicians to regulate the lifestyles, diets, and physical and social activities of patients [3,34,39]. Limitations of conventional treatment approaches in AD are given in Table 1.

Chronic diseases seen in the elderly population are multifactorial in most cases, and even if they are due to a single cause, the use of multiple drugs is usually required for treatment. Depending on pharmacodynamic and pharmacokinetic changes with aging, the rates of absorption, distribution, and excretion of drugs in the body vary considerably. Adverse effects may be exacerbated due to drug interactions due to polypharmacy (concurrent prescribing of at least four or five drugs). This situation reduces patient compliance and causes delays in treatment [90]. For these reasons, there is a need for new-generation therapeutic compounds and drug molecules with a wide therapeutic index and low side effects in AD, where disease-modifying therapy is incomprehensible and difficult [54].

Instead of single-target anti-AD drugs in the treatment of AD, it has been adopted to administer more than one targeted drug at the same time. These multiple targets are AChE, metal ions, and free radicals. Drugs, which can inhibit the AChE enzyme, chelate metal ions, and capture free radicals at the same time, are becoming much more interesting [91].

In a review, it was mentioned that grape polyphenols were the polyphenols in grapes that attenuated some AD pathophysiological processes, including the formation of Aβ plaques and neurofibrillary tangles, oxidative stress and inflammation, and synaptic dysfunction [92]. Rosmarinic acid, which is abundant in plants, such as rosemary and basil, reduces the accumulation of Aβ [93,94].

## 6. Novel Phyto-Therapeutic Approaches in AD

Phyto bioactive compounds are plant-based ingredients that contain pharmacologically active molecules and act at various target sites, also known as ‘herbal medicinal’ products. These plant-derived compounds are superior to synthetic drug molecules in many respects, due to their capacity to bind to a wide variety of target sites in cells and tissues, having a large number of voids in their structures, and the ease in their production [88,93]. In terms of experimental and drug production processes in the laboratory, the active ingredients of herbal-based products can be isolated in high amounts since they are obtained from nature. This provides experimental and production convenience when designing drug formulations [93].

AChEI, which is most often used in the symptomatic treatment of AD, causes side effects, such as nausea, vomiting, and appetite disorders, due to intense bowel movements [16]. Moreover, complications related to the frequency of drug interactions, such as cognitive disorders, dizziness, drowsiness, and delirium, due to polypharmacy in the elderly, have started to become serious [88]. On the other hand, phytopharmaceuticals, their safety, and fewer side effects can make these medicinal products an ideal option, especially in patients struggling with more than one chronic disease.

The balance of ions, such as Cu^2+^, Zn^2+^, and Fe^2+^ in the brain is very important in maintaining physiological processes [95,96]. The imbalance of these ions induces pathologies caused by Aβ and tau and exacerbates the misdistribution/accumulation of metal ions. Therefore, the imbalance of these ions can associate with various neurodegenerative diseases such as AD [96]. In this context, it is known that some polyphenols have an affinity for binding Fe^2+^ ions. Tannic acid (TA) chelates with Fe^2+^ ions [97]. Depending on the concentration, other phenolic compounds such as catechin molecules also chelate with Fe^2+^ ions in the aqueous environments [98]. Moreover, certain plant derivatives have been found to contain essential minerals with metal ions such as calcium, potassium, iron, sodium, zinc, and magnesium, which are vital to maintaining the body’s fluid and electrolyte balance [99,100].

The use and mechanism of action of many herbal products and phytochemicals in the treatment of AD have been studied in various animal models and clinical studies. Some of these products show promise in the treatment of neurodegenerative diseases with their neuroprotective, antioxidant, anti-inflammatory, anti-amyloidogenic, and anticholinesterase activities [101].

### 6.1. The Effect of Resveratrol on AD

The anti-amyloidogenic effect of different polyphenols in wine was investigated in cell culture, and resveratrol was proven to reduce Aβ levels significantly with increasing concentrations [102]. The neuroprotective effects of resveratrol against Aß-related oxidative stress were also demonstrated [103]. The decrease in the risk of AD development may be due to the antioxidant and neuroprotective properties of resveratrol, a polyphenol abundant in wine.

Polyphenols have been shown to be effective in the pathways involved in the pathogenesis of AD [104,105]. One of these mechanisms is sirtuins (SIRT1-7 in mammals), which are one of the molecular pathways that regulate aging. Sirtuins are regulated by the NAD + /NADH ratio (thus maintaining cellular energy balance) and are important in energy metabolism, circadian rhythm, and aging [106]. Regarding AD, SIRT1 overexpression in the brain activates αsecretase, inhibiting amyloid precursor protein (APP) cleavage and reducing Aβ plaques and amyloid aggregation. It also reduces neurofibrillary tangle formation by deacetylation of tau. Polyphenols can specifically modulate SIRT1-7 to anti-Alzheimer’s activity [104]. Sirtuins can affect ROS production and increase resistance to the harmful effects of oxidative stress, while also being affected by oxidative stress. [107]. Resveratrol has been shown to prevent neurodegeneration, such as amyloid load and tau hyperphosphorylation, with SIRT1 activation and reduce cognitive impairment in AD [108]. Studies support that the use of Resveratrol, which protects against β-amyloid plaque formation and oxidative stress, can delay the onset of neurodegenerative disease, or alleviate the disease [107,109].

In addition, in the rat model of AD, significant down-modulation of Aβ levels was detected in rats treated with resveratrol before the induction of AD than in rats treated with resveratrol after AD induction, and these effects were comparable to rats with AD given memantine. This study demonstrated both the therapeutic and AD protective potential of resveratrol against AD [109]. Resveratrol, an early-stage AD rat model, exhibited neuroprotective effects by reducing oxidative stress and protecting the brain against memory impairment and hippocampal damage. This research showed that resveratrol may be a treatment option for patients with hypertension who are at risk of AD in old age [110]. The protective effects of resveratrol on neuroinflammation and cognitive deficits induced by lipopolysaccharide (LPS) in rats were examined, and the potential of resveratrol to be used in neuroinflammation-related diseases, such as AD, was emphasized [111].

There is strong evidence that resveratrol is effective in animal models of AD and in vitro studies. A randomized, placebo-controlled, double-blind, multicenter, and 52-week phase 2 clinical trial of resveratrol was conducted to investigate the safety, tolerability, efficacy, and side effects of resveratrol in individuals with mild to moderate AD. While there was a significant decrease in cerebrospinal fluid (CSF) Aβ40 and plasma Aβ40 levels in volunteers using resveratrol, there was no significant change in other AD biomarkers. Resveratrol use was reported to be safe and well-tolerated, but increased brain volume loss [112]. The bioavailability of oral resveratrol was poor; and it was shown that it could be detected in the brain by crossing the blood–brain barrier at low concentrations [113]. In the phase 2 study by Turner et al., the pharmacokinetics of oral resveratrol were determined, CSF transfer was specified, and it was administered at the highest well-tolerated dose [105]. In another study, while cognitive functions decreased in the placebo group, it was shown that high-dose resveratrol treatment could slow down progressive cognitive and functional decline by reducing neuroinflammation [114]. In a clinical study examining the progression of AD with the use of low-dose oral resveratrol, it was determined that there was less deterioration in cognitive functions in the use of preparations containing resveratrol glucose and malate compared to placebo and that this change was, however, not statistically significant [115]. Many studies were conducted in vivo and in vitro models to investigate the therapeutic efficacy of resveratrol in the treatment of AD. However, these studies are still insufficient to describe the efficacy and to elucidate the mechanisms, and more research is needed.

### 6.2. The Effect of Tannic Acid on AD

Tannic acid (TA) is a well-known hydroxybenzoic acid. It consists of 10 gallic acids bound to glucose [116]. Cell culture experiments showed that the effects of red wine-derived polyphenols on AD were due not only to their antioxidant properties but also to anti-amyloidogenic activities. [117,118]. Ono et al. revealed that polyphenols inhibited the formation of beta-amyloid fibrils (fAβ) from Aβ and also destabilized the preformed fAβ [98,110]. From these studies, it was thought that the more hydroxyl groups were in the molecule, the higher the anti-amyloidogenic activity could be. The anti-amyloidogenic activities of TA, which is a plant-derived tannin with high hydrolyzable polyphenol content, were investigated. It was determined that tannic acid had a stronger anti-amyloidogenic and fibril-destabilizing activity than NDGA, and that it may be a key molecule in the treatment of diseases such as AD [118].

Orally administered TA improved cognitive functions and prevented behavioral deterioration in a transgenic mouse cerebral amyloidosis model for six. It was determined that TA application alleviated β-amyloid deposits and AD pathology by inhibiting β-secretase activity and neuroinflammation [119]. A Sporadic AD (SAD) model was created by intracerebroventricular streptozotocin (icvSTZ) injection to rats pretreated with TA for 21 days. Learning and memory disorders increased AChE activity, and oxidative stress was observed in rats treated with STZ only. However, it was shown that TA could prevent the AChE increase induced by STZ, most of the neurochemical changes and oxidative damage, improve behavioral changes, and prevent learning and memory deterioration. The protective effects of TA against AD were demonstrated in some in vitro and in vivo transgenic AD models. [118,119,120]. When compared with gallic acid (GA), GA did not have a similar effect due to its structural differences [120]. It was stated that TA may be a leading compound of anti-AD drugs, that it was probably difficult to cross the blood–brain barrier directly due to the polar structure of the TA molecule, and that it could, however, pass through transport systems.

### 6.3. The Effect of Apigenin on AD

Neuroprotective and anti-inflammatory effects of apigenin were demonstrated in vitro in the AD-related neuroinflammation model, emphasizing that it may be an important neuroimmunomodulator in the treatment of AD. [121]. When AD neurons generated using the human-induced pluripotent stem cell (iPSC) model of familial and sporadic AD were compared with healthy control neurons, neuronal hyper-excitability and Ca^2^+ dysfunction, increased cytotoxicity, apoptosis, and inflammation, increased nitrite levels, and reduced neurite length were demonstrated. Apigenin in AD neurons was shown to reduce neuronal hyper-excitability and apoptosis, as well as protect from neuronal damage and death with its anti-inflammatory activity [122]. In addition to its antioxidant, anti-inflammatory, and neuroprotective properties, apigenin could inhibit anti-Aβ aggregations and cholinesterase. It was reported that the newly synthesized compound performed selective metal chelation and ameliorates AlCl_3_-induced zebrafish AD and scopolamine-induced memory impairment. [123]. In a study on the effect of apigenin on the AD mouse model, apigenin alleviated the Aβ load, suppressed the amyloidogenic process, inhibited oxidative stress, and improved AD-related learning and memory impairment [124].

### 6.4. The Effect of Psoralea corylifolia L. on AD

The chemical composition of Psoraleae of Psoraleae Fructus, the dried fruits of *Psoralea corylifolia* L., contains bavachin, bavachinin, bavachalcone, and isobavachalcone compounds [125]. Compounds from Psoraleae Fructus were able to detect neuroinflammation, oxidative damage and amyloid β-peptide 42(Aβ42), BACE1(β-secretase) activity, glycogen synthase kinase 3β, and AChE, which play a role in the basic mechanisms of AD, in vitro. It was shown to inhibit it in different ways. Bavachalcone was shown to have the potential to be converted into a powerful inhibitor, such as curcumin, which is a well-known neuroprotective agent on neurodamage and spontaneous Aβ42 aggregation, and it was emphasized that its potential clinical values in AD treatment needed further research in vivo [126].

Compounds isolated from Psoralea Fructus (PF) were shown to strongly inhibit Aβ42 aggregation by using molecular simulation modeling by computer simulations [127].

Senescence-accelerated mouse-prone 8 (SAMP8) is an ideal model to study AD. SAMP8 demonstrates age-related learning and memory impairments and allows the examination of many features of AD pathogenesis, including oxidative stress, inflammation, Aβ deposits, and tau hyperphosphorylation [128]. Anti-Alzheimer’s effects were compared with resveratrol, which is used as a neuroprotective compound, by administering to SAMP8 mice total prenylflavonoid (TPFB) prepared from dried fruits of *Psoralea corylifolia* L. TPFB is a dietary supplement with eight major components: bavachalcone, isobavachalcone, neobavaisoflavone, bavachin, bavachinin, corylin, psoralidin, and corylifol. Long-term administration of TPFB significantly improved cognitive performance and exhibited similar responses to resveratrol in Morris water maze tests). It was found that TPFB significantly reduced the level of Aβ42 and inhibited hyperphosphorylation and reduced proinflammatory expression and oxidative stress [129]. It was stated that prenilflavonoids obtained from *Psoralea corylifolia* L. are promising drug candidates, especially bavachalcone, which prevents AD-like neurobiochemical changes [129,130,131].

### 6.5. The Role of Curcuminı in AD and Preventatıon

A hydrophobic natural pigment, curcumin is also known as turmeric. It is found in the underground roots of the plant, namely, the rhizomes [132]. Curcumin was shown in cell culture to prevent oxidant damage with its antioxidant activity, regulate the cellular signaling pathway and prevent Aβ from forming β-layer aggregation and neurotoxicity [133]. Chronic inflammation is especially important in the early phase of AD. The anti-inflammatory and antioxidant properties of curcumin were able to reverse neurotoxicity by affecting Aβ accumulation in the mouse AD model [134]. It was reported that curcumin modulated neuroinflammation, regulated multiple cellular signaling pathways, and reduced oxidative damage [135,136]. It was shown that chronic curcumin treatment in rats with chronic aluminum toxicity reduced oxidative damage and AChE activity and improved cognitive dysfunction [137]. In another study, in an animal model of streptozotocin icvSTZ-induced SAD, curcumin was shown to ameliorate impaired insulin/IGF signaling, reduce neuronal damage, and enhance cognitive ability [138]. In a 48-week placebo-controlled phase 2 study in 36 volunteers with mild to moderate AD, curcumin levels could not be detected in plasma and CSF, suggesting limited bioavailability of curcumin. Clinical or biochemical evidence of curcumin’s efficacy could not be demonstrated due to insufficient sample size and short study duration [139]. Similarly, the efficacy of curcumin used in clinical and cognitive measurements could not be demonstrated in a clinical study conducted on 96 patients for 12 months [140]. In observational clinical research, it was stated that increasing dietary curcumin consumption played an important role in reducing the risks of healthy cognitive aging and dementia [141].

### 6.6. The Effect of Rutin on AD

Rutin was shown to inhibit Aβ aggregation and cytotoxicity, reduce oxidative damage, and reduce the production of nitric oxide and proinflammatory cytokines in vitro, with anti-inflammatory activity [142] However, to investigate the protection of rutin against AD, it was reported that rutin reduced Aβ load, oxidative stress, and neuroinflammation, and significantly improved memory deficits in the AD transgenic mouse model [143]. Rutin was converted to Sodium rutin (NaR), which provides high water solubility and bioavailability, and NaR treatment was shown to restore microglial phagocytic capacity and promote Aβ clearance, reduce neuroinflammation, and improve learning and memory deficits in AD mice [144].

### 6.7. The Role of Quercetın in Treatment and Preventatıon of AD

Quercetin treatment (aluminum chloride-induced) was proven to be a protective role by reducing oxidative stress, inhibiting AChE, and increasing cognitive and behavioral functions in a zebrafish AD model [145]. In an aluminum chloride-induced AD rat model, treatment with quercetin nanoparticles (QNPs) prevented the formation of NFTs and amyloid plaques, increased tyrosine hydroxylase (TH) activity, and thus improved neuronal function. It was stated that quercetin nanoparticles could be used to prevent or delay the onset of AD [146]. In transgenic AD model mice, quercetin reduced Aβ-mediated cytotoxicity, tauopathy, and histopathological symptoms, and improved cognitive and emotional impairments without adverse effects [147]. Quercetin has beneficial properties against mechanisms of AD pathology, as shown in various in vitro and in vivo models. It was shown to reduce oxidative stress and neuroinflammation, inhibit Aβ aggregation and tau hyperphosphorylation, and restore acetylcholine levels with AChE inhibition. Although it had neuroprotective activity in these AD models, its low bioavailability and low brain penetration limit its use in neurodegenerative disorders [148].

Newly developed senolytic agents are drugs that selectively induce apoptosis of senescent cells, and the combination of dasatinib (D) and quercetin (Q) (D + Q) is one of them. D + Q treatment was shown to reduce serum inflammatory mediators and attenuate age-related cognitive dysfunction when aged rats are given D + Q treatment [149]. Senolytic treatment of AD mice D + Q was shown to selectively clear Aβ plaque environment senescent cells, reduce Aβ burden, reduce neuroinflammation, and improve cognitive deficits [150]. Combination therapy of senolytics, dasatinib, and quercetin was thought to be useful for aging-related diseases, and for early-stage AD. A first open-label phase 2 clinical trial was initiated, demonstrating an acceptable safety profile [151].

### 6.8. The Effect of Caffeic Acid on AD

Caffeic acid inhibited Aβ aggregation and disrupted mature fibrils in both aqueous and cellular lipid membrane-like environments [152]. Administration of caffeic acid phenethyl ester (CAPE) in the AD mouse model improved learning and memory as well as antioxidant, anti-apoptotic, and anti-inflammatory activity [153]. Pre-treatment with caffeic acid (CA) and caffeic acid phenethyl ester (CAPE) to mice (AH) in whom the AD model was created by the administration of acrolein was shown to significantly reduce acrolein-induced oxidative stress, neurotoxicity, and GSH depletion. In addition, it was stated that CA/CAPE regulated α-secretase and β-secretase (BACE-1) enzyme activation induced by acrolein and could be used in the treatment of neurodegenerative diseases [154]. In the icvSTZ AD model, the cholinergic activity, antioxidant, anti-inflammatory, and memory-improving functions of CAPE treatment in the brain were inhibited when PI3-kinase inhibitor was administered to rats. In the same experiment, inhibition of eNOS by administration of L-NAME weakened the anti-AD effects of CAPE. These results revealed that the brain PI3-kinase activity of CAPE and eNOS-mediated NO transmission played a role in neuroprotection and memory improvement [155,156]. In a Cardiovascular Risk Factors, Aging, and Dementia (CAIDE) study, drinking 3–5 cups of caffeine/coffee a day in middle age reduced the risk of dementia/AD/cognitive functioning coffee in older age by approximately 65%. It was stated that this effect may be mediated by mechanisms such as the antioxidant effect of caffeine and increased insulin sensitivity [157].

### 6.9. The Effect of Hesperidin on AD

Citrus fruits have different kinds of polyphenolic molecules such as hesperidin, hesperetin, naringin, limonene, naringenin, neohesperidin, poncirin, and neoeriocitrin [158]. In the transgenic AD mouse model, the application of Hesperidin for 16 weeks improved total antioxidant capacity. Although it did not affect Aβ accumulation, it ameliorated mitochondrial dysfunction and improved cognitive functions and locomotor activity [159]. Interestingly, in the aluminum chloride-induced AD model, Hesperidin was found to reduce AChE activity, suppress Aβ accumulation by suppressing β- and γ-secretase expression, and reverse learning and memory impairments [160]. In the icvSTZ sporadic AD model, hesperidin showed neuroprotective activity by modulating AChE activity and lipid peroxidation and blocking the inflammatory process, and improving cognitive deficits and memory [161]. Despite various animal-based studies on the role of hesperidin in the treatment of AD, there are no clinical studies to elucidate the neuroprotective properties and mechanisms of this phytochemical in Alzheimer’s patients. In a placebo-controlled, randomized, and double-blind clinical study examining the dose-dependent effect of chronic orange juice consumption in healthy older adults, it was determined that the cognitive and executive functions of the group consuming orange juice with high hesperidin content were better than other groups [162]. Clinical studies showed that hesperidin-enriched dietary supplements increase cerebral blood flow and significantly improved cognitive function and memory performance in healthy subjects [162,163].

The effect of citrus consumption on dementia was examined in an observational study involving Japanese elderly volunteers. It was determined that the incidence of dementia and citrus consumption of the participants showed a statistically significant inverse correlation. The possibility and protective role of citrus flavonoids in reducing the risk of dementia was demonstrated [164]. However, despite the variety of in vivo studies on hesperidin, the lack of clinical studies on the neuroprotective activity, AD therapeutic effects, bioavailability, appropriate dose, and tolerability of hesperidin limits the use of hesperidin.

### 6.10. The Effect of Limonene on AD

The antiproliferative, apoptosis-inducing, and antioxidant effects of D-Limonene, a common monoterpene, were demonstrated [165]. In an animal (fly) model of AD, it was stated that limonene had antioxidant and anti-inflammatory properties and a neuroprotective effect on Aβ cytotoxicity; however, its mechanism was not clarified [166].

### 6.11. The Effect of Berberine on AD

Berberine (BBR) showed therapeutic efficacy against damaged nerve cells; so, it was suggested for nerve repair. BBR’s small size and ability to cross the blood–brain barrier made it the agent of investigation for AD [167]. The therapeutic effect of berberine on neuropathology and cognitive impairment was evaluated in a transgenic AD mouse model. With BBR treatment, the modulation of pathological processes, such as β-amyloid levels and tau hyperphosphorylation and amyloid plaque accumulation, decreased, and cognitive functions improved [168]. In studies using mouse models of AD, it was reported that berberine had antioxidant and anti-inflammatory activities as potential mechanisms underlying its anti-AD property, as well as inhibition of AChE activity and anti-amyloid effects, improving cognitive functions [169]. In addition, berberine inhibited Aβ production by reducing BACE1 protein levels in the brains of AD mouse models [170]. The low bioavailability of this natural product, which has a wide range of pharmacological effects, was stated to be the biggest obstacle to its pharmaceutical development [169]. Safety evaluation and clinical studies of berberine are needed to clarify its role in AD-related pathologies such as BBR, limitation of extracellular amyloid plaques, and intracellular NFTs [171].

### 6.12. The Effect of Cinnamon on AD

Cinnamon, cinnamon polyphenols, and cinnamaldehyde obtained from the inner bark of trees of the genus Cinnamomum were traditionally used in the treatment and prevention of AD onset and/or progression by suppressing oxidative stress and proinflammatory signaling pathways in the brain [172]. Cinnamon extract administered to a fly model of AD was shown to inhibit the formation of amyloid fibrils and lead to improvement in cognitive behavior. In the same study, it was noted that cinnamon extract was able to cross the blood–brain barrier, reduce plaque formation, and improve cognitive behavior in an AD transgenic mouse model. PC12 preserved cell viability by dose-dependent inhibition of the cytotoxic effect of Aβ fibrils. Cinnamon extract, which was studied in vitro and in vivo, had good oral bioavailability and was a unique multi-component herbal supplement that could be used safely in the treatment of AD [173].

Decreased expression of the insulin receptor and insulin signaling pathway was noted in impaired brain function and Alzheimer’s patients and AD animals. In a non-transgenic AD mouse model, CE administration was reported to increase GSK3β, which improves insulin signaling, inhibits AChE activity, and improves learning skills [174]. Cinnamic acid derivatives have various pharmacological activities such as anti-inflammatory, antioxidant, neuroprotective, and anti-amyloid aggregation activities. The cinnamic acid–tryptamine hybrid series was designed and synthesized and demonstrated AChE inhibitory effects for the treatment of AD [175].

### 6.13. The Effect of Ginger on AD

Ginger extracts’ inhibition of AChE activities in the brain and prevention of lipid peroxidation are the possible mechanisms of their anti-Alzheimer properties [176]. Molecular models and calculations were made on the components of ginger, which were reported as AChE inhibitors in the brains of AD patients, and it was stated that it could be preferred in the design of new inhibitors compared to donepezil [177]. When the protective and therapeutic effect of ginger in AD was investigated in the AlCl_3_-induced AD model, it was found that long-term ginger treatment had curative properties, and that short-term use did not, however, provide significant improvements [178,179].

### 6.14. The Role of Luteolin in AD Treatment

Carotenoids provide neuroprotection by attenuating neuro-inflammation and oxidative damage, activation of antioxidant systems, modulation of autophagy, and microglial activation [180]. It is thought that carotenoids, such as lutein, zeaxanthin, lycopene, α-carotene, β-carotene, and β-cryptoxanthin, can be used in the treatment of AD thanks to their antioxidant and anti-inflammatory properties in the brain [181,182,183].

Within the scope of the Rush Memory and Aging Project, elderly people without AD were observed for a long time, and the postmortem brain autopsies of some participants were examined to evaluate the relationship between individual carotenoid intake and AD risk, and the underlying neuropathological basis was investigated. It was reported that total carotenoid consumption, especially lutein/zeaxanthin, may have a beneficial role on the incidence of AD, related to inhibition of brain β-amyloid accumulation and fibril formation [184]. In the clinical research, in which postmortem brain tissues were also examined, an association was found between serum lutein, zeaxanthin, and β-carotene concentrations or lutein concentrations in the brain and age-related cognitive performance [181]. The strong antioxidant and anti-inflammatory activity of luteolin were investigated for use in the treatment of neurodegenerative diseases [185]. Chronic cerebral hypoperfusion is associated with cognitive deficits in AD, and the effects of luteolin use on neurocognitive pathologies were investigated in rats with permanent bilateral carotid artery occlusion. Luteolin significantly cleared ROS, reduced lipid peroxidation, and suppressed the inflammatory reaction. However, long-term luteolin administration was found to reduce BACE1 inhibition and Aβ accumulation and improve cognitive functions [186]. It was shown that the ability of luteolin to inhibit AChE, and BACE1 was greater than the inhibitory activity of orientin and isoorientin [187].

In the fly model (*Drosophila*) of AD, a dose-related increase in lifespan and a delay in dexterity were observed for rat-administered luteolin. Luteolin oxidative stress decreased AChE activity and prevented Aβ plaque formation [188]. It was stated that luteolin was a potential candidate for the treatment of AD in the icvSTZ AD model, where it exhibited antioxidative properties and neuroprotective properties by dispersing Aβ plaques, improving memory impairment [189].

In the review on the risk of AD development, it was stated that the findings obtained from observational studies did not protect the habitual intake of vitamin C, vitamin E, β-carotene, or flavonoids from the development of AD [190]. Innovative technologies, including artificial intelligence and deep learning, and unique multidimensional and personalized designs should be considered as much as possible in clinical research to help resolve the multifactorial nature of AD [183].

In the literature, the mechanism of the protective action of phytocompounds such as resveratrol, TA, apigenin, curcumin, quercetin, etc., on the nervous system is extensively investigated and some of them are summarized in Table 2 [191,192,193,194,195,196,197,198,199,200].

As can be seen from Table 2, the plant-based compounds provide therapeutic effects in AD and AD-related dementia through different mechanisms such as attenuating neuroinflammation, inhibiting AChE, delaying Aβ plaque formation, reducing and improving oxidative damage, and preventing neurotoxicity-related complications.

### 6.15. Potential Effects of Ginkgo biloba in AD Treatment and Prevention

The extract obtained from the leaves of the *Ginkgo biloba* (GB) plant improves cerebral and peripheral circulation, especially in the elderly, and can be used as a folk remedy for complaints such as vertigo and tinnitus [201]. The components known to be responsible for the activity in the plant extract are flavonoids and terpene lactones [201]. In one of the earliest randomized controlled trials (1997) on the use of GB in dementia, cognitive improvement was seen in a remarkable majority of various tests such as AD Assessment Scale-Cognitive subscale (ADAS-Cog), Clinical Global Impression of Change, and Geriatric Evaluation by Relative’s Rating Instrument (GERRI) [202]. The effect of GB extract on dementia patients with severe neuropsychiatric symptoms was studied, and in two 22-week trials, the extract-treated group showed superiority over the control [203]. In a recent meta-analysis study, GB extract was found to significantly improve behavioral and psychological symptoms of dementia, other than psychotic-like features, and caregiver distress score [204]. Daily intake of GB up to 240 mg did not cause any effect on the activities of elements of the Cytochrome P450 mixed-function oxidase system [201]. It was added that various changes in activity might occur depending on the number of unwanted components such as biflavone, gincolic acid, and the actually wanted terpene lactones in the extract [201,204]. In addition, the combination containing the extracts of Panax ginseng (*P. ginseng)*, which is known to have a synergistic effect with GB [205], was tested on more than 60 individuals with symptoms of forgetfulness and a high degree of compliance with the diagnostic criteria. After 4 weeks of treatment with this *P. ginseng*–GB combination preparation, a high rate of improvement in the Mini-Mental State Examination score was detected [206]. GB extract acts at the tissue level by providing membrane stabilization, inhibiting lipid peroxidation, and enhancing tissue perfusion in patients with radiation-induced cognitive impairment [207]. According to the phase II trial results in this study, it was thought that 120 mg of GB extract daily could improve the memory and quality of life of patients undergoing brain irradiation [207].

Different formulations of Ginseng compositions patented for Alzheimer’s disease, dementia, and cognitive disorders, and some of them along with their details are given in Table 3 [208,209,210].

From some of the patented studies given in Table 3, various ginseng compounds with different chemical compositions and formulations are utilized as health-promoting material or suggested for healing potential in AD and AD-related dementia with selective enzyme inhibition, improvement of cognitive damage, regulation of neuron metabolism, and nucleic acid regulation.

## 7. Conclusions and Perspectives

The pathogenesis of AD is closely related to cholinergic dysfunction, mitochondrial abnormalities, neuroinflammation, and oxidative stress in the CNS and eventually results in cognitive decline and memory impairment. Efforts are being made to develop effective tools for the early diagnosis and successful treatment of this progressive neurodegenerative disease. Symptomatic therapy of AD includes AChE inhibitors (donepezil, rivastigmine, etc.), NMDA antagonists (Memantine), anti-inflammatory drugs (rofecoxib), secretase inhibitors (Memoquin), and anti-Aβ immunotherapy (Aducanumab). However, the efficacy of these drugs is limited due to the intricate pathogenesis of AD. Considering inadequate drug response in patients, low patient compliance, as well as the significant side effects, the subject of the research is globally directed to herbal products with their excellent properties such as high biocompatibility, highly tolerable nature, non-toxicity, causing fewer side effects, lower price, and controllable bio-distribution in the human body.

In this review, neuropharmacological mechanisms are discussed for phytochemicals that have preventive and therapeutic effects in Alzheimer’s disease, which is one of the neurodegenerative diseases with high prevalence and incidence. The neuroprotective mechanism of natural products used for treatment in various cell and animal experimental models specific to AD is thought to be mediated by the improvement of endogenous antioxidant defense functions and the inhibition of neuro-inflammatory and apoptotic pathways. Despite several preclinical studies of these molecules, large-scale clinical trials based on voluntary participation are needed to confirm their neuroprotective efficacy in humans and to evaluate their safety profile.

## Figures and Tables

**Table 1:** Limitations of conventional treatment approaches in AD.

Current Treatment Modalities in Alzheimer’s Disease	Limitations and Failures in Treatment
Acetylcholinesterase Inhibitors	Not effective in all patient groups, may cause intense gastrointestinal side effects, drowsiness, insomnia, heart rhythm disorders
Memantine	Clinical efficacy differs in moderate-to-severe AD and mild AD There are insufficient data on the long-term safety and benefit of starting memantine therapy early
Immunotherapy	The definitive results of the use of immunotherapy in AD have not been disclosed and certain immunization assessments could not be tested in humans
Antipsychotics and antidepressants	Some of this group of drugs can exacerbate AD sequelae Treatment may be interrupted due to drug interactions Only moderate results can be obtained with antipsychotic group drugs
Statins	Only certain lipid-lowering drugs can give results in treatment
Vitamin supplements	Antioxidant supplements have not been associated with reduced incidence of dementia in asymptomatic individuals

**Table 2:** The neuroprotective effects of phytocompounds on AD.

Phytocompounds	Mechanism of Action	Reference
Resveratrol	Reduces neuroinflammation, inhibits tauopathy and Aβ-plaque formation, decreases the level of SIRT1 levels in neurons, prevents NF-κB activation, reduces the activity of β-secretase and reduces oxidative stress in brain, prevents apoptosis	[191]
Tannic acid	Natural inhibitor of β-secretase (BACE1) activity, destabilizes neurotoxic Aβ fibrils, inhibits the aggregation of tau peptide and NFTs	[192]
Apigenin	Regulates the expression of inflammatory mediators in neurons/glial cells, protects neuroinflammation, reduces Aβ, fibrillar amyloid deposits, oxidative stress, and neuronal hyper-excitability	[193]
Curcumin	Improves neuronal apoptosis, restores cerebral blood flow, and reduces AchE activity, suppresses tau aggregation, reduces oxidative stress	[194]
Quercetin	Inhibition of Aβ aggregation and tau phosphorylation, inhibits (BACE-1) enzyme activity, competitively inhibits AChE, modulates the cell’s own antioxidant pathways	[195]
Hesperidin	Attenuates APP expression and suppresses the levels of Aβ and β- and γ-secretase activity, decreases AChE activity and lipid peroxidation, blocks inflammatory process, increases the anti-oxidative defense system, diminish neuro-inflammatory and apoptotic pathways	[196]
Limonene	Decreases AChE activity, reduces oxidative stress	[197]
Berberine	Reduces Aβ levels, inhibits BACE-1 activity, decreases the hyperphosphorylation of tau. Berberine retards oxidative stress and neuroinflammation in the brain	[198]
Cinnamon	Inhibits the formation, accumulation, and toxic effects of Aβ plaques, and has potential antioxidant effects and restoration of redox balance	[199]
Ginger	Inhibits AChE activity, reduces Aβ levels and β- and γ-secretase activity, represses neuroinflammation and amyloid genesis, acts as a radical scavenger, prevents apoptosis	[200]

**Table 3:** Ginseng compositions patented for Alzheimer’s disease and cognitive disorders.

Development Status (Description/Study Design)	Chemical Constituents	Mechanism of Action (Outcomes)	Patent Number	Reference
3 monthly age APP695V717I transgenic mice donepezil hydrochloride control group and Chinese medicine group orally for 6 months	Traditional Chinese medicine: Prepared fleece flower, ginseng, *Rhizoma Acori Graminei*, Coptis and Chuanxiong Rhizome	This medicine improves the impaired ability of learning and memory of APP transgenic mice, improves the content of Ach, strengthens the activity of choline acetyltransferase (CHAT), suppresses the activity of AchE	CN102078460	[208]
Wistar senile rats’ donepezil hydrochloride control group and Chinese medicine group orally once a day, continuous for 8 weeks		This medicine improves space learning memory ability of old cognitive disorder rats, alleviates hippocampus neuronal damage, by influencing lipid metabolism, oxidative stress, level of inflammation and apoptosis (Aβ level, bcl-2 expresses, NF-KB)		
Cognitive disorder model mouse	The traditional Chinese medicine composition: thin leaf milkwort rootbark, sweet flag rhizome, ginseng, Gastralia Tuber, paper mulberry fruit, ginkgo leaves, and borneol	This medicine can significantly improve the learning memory central brain SOD activity, reduce its neuronal cell lipofuscin content, and oxygen-free radical injury	CN105943888A	[209]
Four-vessel occlusion-induced cerebral ischemia rat models	Ginseng mixed herbal extracts, ginsenoside Rg2, and ginsenoside F2	This medicine improves memory capacity of symptoms such as mild cognitive impairment or dementia, and shows excellent acetylcholinesterase inhibitory activity and antioxidant activity	KR101509056B1	[210]

## Data Availability

Not applicable.
